# Clinical Holistic Medicine: A Psychological Theory of Dependency to Improve Quality of Life

**DOI:** 10.1100/tsw.2004.124

**Published:** 2004-08-13

**Authors:** Søren Ventegodt, Mohammed Morad, Isack Kandel, Joav Merrick

**Affiliations:** ^1^ The Quality of Life Research Center, Teglgåstraede 4-8, DK-1452 Copenhagen K, Denmark; ^2^ Division of Community Health, Ben Gurion University, Beer-Sheva, Israel; ^3^ Faculty of Social Science, Department of Behavioral Sciences, Academic College of Judea and Samaria, Ariel, Israel; ^4^ National Institute of Child Health and Human Development, Office of the Medical Director, Division for Mental Retardation, Ministry of Social Affairs, Jerusalem and Zusman Child Development Center, Division of Pediatrics, Ben Gurion University, Beer-Sheva, Israel

**Keywords:** quality of life, QOL, philosophy, human development, holistic medicine, public health, holistic health, holistic process theory, life mission theory, group therapy, Denmark

## Abstract

In this paper, we suggest a psychological theory of dependency as an escape from feeling existential suffering and a poor quality of life. The ways in which human beings escape hidden existential pains are multiple. The wide range of dependency states seems to be the most common escape strategy used. If the patient can be guided into the hidden existential pain to feel, understand, and integrate it, we believe that dependency can be cured. The problem is that the patient must be highly motivated, sufficiently resourceful, and supported to want such a treatment that is inherently painful. Often, the family and surrounding world is suffering more than the dependent person himself, because the pattern of behavior the patient is dependent on makes him or her rather insensitive and unable to feel. If the patient is motivated, resourceful, and trusts his physician, recovery from even a severe state of dependency is not out of reach, if the holistic medical tools are applied wisely. The patient must find hidden resources to take action, then in therapy confront and feel old emotional pain, understand the source and inner logic of it, and finally learn to let go of negative attitudes and beliefs. In this way, the person can be healed and released of the emotional suffering and no longer be a slave to the dependency pattern.

## INTRODUCTION

In his book *Women Who Love Too Much*, Robin Norwood[1] has the outstanding insight that dependency is generally due to an urge to escape from oneself into something else. A dependent person has leaped out of himself or herself, from his or her existence and abdomen, into whatever it is that he or she is dependent on. The purpose of leaping out of oneself is quite simple: to avoid feeling the pain in life; to avoid feeling everything in life that is far removed from the way it ought to be.

The interesting aspect is that many different things can be used to escape — sex, television, work, alcohol, drugs, the lottery, the Internet, other people, and more — resulting in dependency. It is always a habit that provides freedom from pain by diverting attention from your own emotions, body, and existence. The pain the patient is escaping from is always immense suffering in the patient's present-day existence, which is located in his or her repressed side in the internal waste bin, the "sack"[2]. Because of the discomfort involved, the patient cannot be blamed for trying to avoid this apparently meaningless pain.

When the patient, supported by a holistic therapist, takes on the emotional pain and accommodates it, explores it, and gets to know it, he or she discovers that there is actual meaning to it. The pain conceals something that we need to learn if we are to become whole people. The dependency is, according to this understanding, not a product of genetic predispositions, although there could be a genetic component also, but a product of a personal history with intensive and repressed existential suffering. Dependency can be cured if this existential pain is integrated and the existence healed. This is the general purpose of holistic medicine.

## THE BASIS FOR CLINICAL HOLISTIC MEDICINE

The life mission theory[3,4,5,6,7,8] claims that everybody has a purpose of life or a huge talent. Happiness comes from living this purpose and succeeding in expressing the core talent in life. Existential pain comes from not being able to live your life purpose. Often this pain is unbearable and therefore repressed. To heal this pain, one must develop as a person into what is known as the natural condition, a condition where the person knows himself by using all his efforts to achieve what is most important for him. The holistic process theory of healing[9,10,11,12] and the related quality of life theories[13,14,15] state that the return to the natural state of being is only possible when the person gets the resources needed for existential healing. The resources needed are "holding" in the dimensions awareness, respect, care, acknowledgment, and acceptance with support and processing in the dimensions feeling, understanding, and letting go of negative attitudes and beliefs. The preconditions for the holistic healing to take place are trust and the intent for healing to take place. Sufficient joy to balance the existential pain is another important prerequisite. Existential healing is not a local healing of any tissue, but a healing of the wholeness of the person, making him much more coherent, resourceful, loving, and knowledgeable of himself and his own needs, nature, and wishes. In letting go of negative attitudes and beliefs, the person returns to a more responsible existential position and an improved quality of life. The philosophical change of the person healing is often a change towards preferring difficult problems and challenges, instead of avoiding difficulties in life and escaping[16,17,18,19,20,21,22,23]. The person who becomes happier, more resourceful, and free often also becomes more healthy, more talented, and more able to function[24,25,26].

## THE MANY FACES OF DEPENDENCY

Hardly anybody can say that they are not dependent on something or somebody. The existential ideal of health with total presence in life with no escape and repression is not of this world. The problem with dependency is not that you spend too much time working or being with your friends or partner or behind the computer, but that you miss your opportunity to live. Many people do not have a life. They are deeply in pain and only because they manage to escape, can they avoid feeling how bad and miserable their life really is. An even more serious problem is the tendency of dependent people to be abusive and violent because they cannot manage the emotional pain when it surfaces, in the moments where their "heroin-like habits" are disturbed. Fortunately, holistic medicine provides us with tools to help the dependent patient integrate his or her life pains and grow from the dependency. But these tools must be used wisely.

> **Female, aged 22 years, with dependency:** First session: The patient presents with a dependency problem – she left her boyfriend a year ago after living with him for three and a half years. She is unable to move on, would like to go back, but that is not possible either. Works 60 hours a week – workaholic – she cannot be alone with herself and her emotions, which appear to be denied. Third eye – appears as though the brain is running on half power – "Am I stupid?" Hara: is not really at home in her stomach, pelvis, and uterus. We work on this on the couch. Heart – appears to be quite closed. Purpose of life: something about being healthy and lovely – right now she is like a flat, lukewarm soda water. EXERCISE 1: One day a week you must do nothing other than be there and live in your emotions. Do not do anything, just be there and feel what you feel. These will be uncomfortable days. EXERCISE 2: Make a complete list of all your problems, describe them on half a sheet of A4 each. About 10 problems. Diagnosis: existential pain, repressed and leaps out of herself and into her boyfriend /dependency/. PLAN: she should come back in 2–4 weeks.
>
> Second session Has been very miserable, but that is a sign that there is progress and the patient is now in contact with her emotions. EXERCISE 1: When you feel something, stop and note what it is that happens. Catch your shadow with your attention. See the little, neglected, and lonely girl in you – study her. Do not change. "I am alone" – is let go of with a roll [the kitchen roll which the hand slowly releases as the statement is released]. EXERCISE 2: When you are together with other people, note what you feel – is there pain in closeness? EXERCISE 3: Describe the good in you, all your talents. Another appointment in 2–3 weeks. Next time – talk about good books.

Here the physician (SV) unfortunately makes the mistake of overburdening the patient with more exercises than she has the resources to cope with. She consequently failed to turn up for the next conversation. It must be acknowledged that the physician in this case was too ambitious on behalf of the patient and consequently deprived her of the chance to receive help.

## THE "CO-DEPENDENT" PERSON IS A KEY PART OF THE PROBLEM

There is often a "co-dependent" person close to the dependent person, who in turn is dependent on the dependent person. The co-dependent person leaps over to the dependent person in the same way that the dependent person leaps out of himself or herself into food, sex, alcohol, work, etc. The co-dependent person is someone who "loves too much", an angel that the severe alcoholic, workaholic, etc. is so fortunate to have by their side and who makes a sacrifice for the other person. But in reality this person is not a victim, but a "criminal". The co-dependent person is not just dependent on being able to help and support the dependent person, but often holds the dependent person fastened in his dependency.

The co-dependent person is an active participant in the person's dependency, but often plays the role so well that the dependent person does not see through the game, but weighed down by the feeling of guilt, reproaches himself for his weak character. A co-dependent person for an alcoholic will ensure, for example, that there is always plenty of alcohol within reach. It is difficult for the dependent person to escape from his dependency when someone close is dependent on the person being dependent! Such complicated correlations may be difficult to see through for the person suffering from dependency, who fails time after time when he tries to give up drinking, to the great dismay of friends and family. There is a need for an outsider with a better overview and understanding of the dynamics of dependency.

> **Male, aged 55 years, female aged 43 years – alcoholic, co-alcoholic:** Quality-of-life conversation – Couple session: They come on her initiative. “We have to change from *I* to *we*,” she says, but I do not agree. She wishes to devour him. She has a long history of being a “co-alcoholic” in a previous relationship, where the man was weak, with alcoholic tendencies. She and her present partner came together last autumn, and now he feels enveloped and half smothered. We talk about this and discover that she plays the role of the “criminal”, while he is the “victim”. EXERCISE for her: Stop saying *we*. There are only two selves here. Stop dominating and “nudging” – that is to say, forcing through your will. Let him take most of the initiatives and let him be in charge when you are together. Let him come onto the stage. Forget yourself. Wait for him. EXERCISE for him: Say no if you feel uncomfortable. Say “it’s private” if there is something you do not want to share. PLAN: Five Rosen sessions for her, so that she can perceive the cause of her need to dominate; session as a couple again with SV in 6–8 weeks.

## THE PATH OUT OF DEPENDENCY IS THE PATH THROUGH PAIN

To become free of abuse, it is necessary to look at the historical life pain within and the present-day life situation at the same time. Becoming free will also mean becoming free in relationships with other people and that is sometimes the scariest aspect, because we do not really need to be dependent on anyone if we can fully and completely count on ourselves. Then we can live, as we want, with those we want. It is only in freedom that love and spirit can flourish.

We have conducted projects with people in severe social distress and with severe dependency, for instance on alcohol[25]. We have also used students to conduct in-depth interviews with substance abusers and prostitutes with drug addictions at the street level. On the face of it, the picture looks bleak for many of them. However, we have been surprised how relatively easy it has been in practice to do something for those of them who wanted to help themselves. Of the alcoholics, 9 out of 16 we have been working with (in an intensive 5-day residential course with a view to improving their life philosophy and life practice) drank less and apparently are leading a much better life, when we examined them 6 months after the course. Three of them had only sporadic episodes of drunkenness and had returned to work[25,26].

The medical consultation for a patient of this type typically comes about as a result of the patient having suffered an acute crisis, which has caused him to reach a decision that now is the time to stop! Our treatment does not, however, always prove successful. The next patient came full of eagerness and enthusiasm and promised firmly that she would improve, but she was never seen again.

> **Female, aged 50 years, alcoholic and heavy smoker, who would like to stop this abuse:** The patient is an alcoholic, who drinks 7–14 units of alcohol daily and smokes 50 cigarettes, but has not been involved in prostitution or drug addiction. Three weeks ago her boyfriend, whom she had known for 17 years, died from drinking. This has stirred things up. There are family problems, as her brother wants her to renounce her inheritance. The patient has been “nice” throughout her life and has done what other people expected of her, but now wishes to become free and alive. INTERPRETATION: It is precisely this attempt to break out that has brought her to the hell – the tormented and death-like state – she now finds herself in and is trying to drink her way out of. When a woman tries to get away from being nice, she goes through the pain it cost to become a nice and disciplined girl to begin with. PLAN: To go from nice to free, the patient has to go through a period of pain and suffering, with all the old failures and decisions to die coming to the surface. As an alcoholic, she can join our program for holistic rehabilitation and would like to have the quality-of-life package [the clinic's 6-month treatment offer]. She goes to see our clinic's gestalt therapist for a treatment and development plan. We expected three phases with this patient: an orientation phase, in which she understands her personal recovery project; a process phase, in which she goes through her old sufferings in therapy and rediscovers her enjoyment of life and hidden resources; and a rehabilitation phase, where she acknowledges her personal purpose of life and has her new life arranged accordingly. The patient is informed of these expectations and that it will be hard work, possibly taking 3 years, but initially 6 months. The patient is a candidate for the growth group [a network group in which patients and people taking the course meet every fortnight to support one another], which will take place in our lecture hall every second Tuesday. AGREEMENT: The patient has 4 weeks to overcome her excessive alcohol and tobacco consumption. It should preferably also be down after 4 weeks. EXERCISE: She has been given quality of life and life philosophy to read. She scales down her alcohol consumption over the next 4 weeks, after which she comes to see the gestalt therapist again.

This patient came a long way in learning about her problems. The next step was to go through old sufferings, which often is the problematic part. Because whatever for? Why should I suffer the hurt? What am I to live for when life just causes pain? This is the reason that alcohol and pills makes it easy to escape. That is why many people in the Western world have excess consumption and dependency problems. Although the outlook appears bleak, we always choose to believe the best about patients who wish to improve their lives. Occasionally, we are positively surprised.

**Male, aged 63 years, and co-alcoholism:**
1. His sciatica is coming along fine.
2. Swollen left leg. On examination there is nothing other than the swelling. No tenderness in the calf, no discoloration or heat. Discontinue Centyl Mite [bendroflumethiazide + potassium chloride] as the patient does not tolerate Centyl. Prescribe Furix [furosemide] 40 mg + KCl.
3. Problems with drinking. His partner is at a loss what to do, it is affecting him greatly. We talk about dependency and about co-alcoholism/co-dependency. EXERCISE: Read *Women Who Love Too Much* (on the topic of dependency)[1].

In the same way that the husband is dependent on alcohol, she is dependent on being the good, great, and strong person who protects and takes care of him (it is typical for the man to cling to the bottle and the wife to cling to her identity as helper/mother/martyr). A single well-chosen book that illustrates the problem is very often all it takes to create awareness and set the wheels in motion.

## DEPENDENCY AS THE KEY PROBLEM IN A RELATIONSHIP

One of the places where dependency is most common and troublesome is in our close and intimate relationships. Some case reports will illustrate this relationship.

**Female, aged 40 years, with dependency on boyfriend:** There have been problems with her boyfriend, whom she has moved in with. She makes sexual demands on him, and he backs off. We talk about her making demands, because she feels that she "has a need" and about the dependency being a manifestation of her escaping from herself instead of being at ease in her own center. Confronted with this, she cries. We talk about being at

> ease and being in balance. EXERCISE: Sit and be where you are. Stop making demands, stop criticizing and controlling. Focus in terms of your thoughts, in terms of your emotions.

Dependency often takes the form of an urge to demand, control, and criticize. When the dependency becomes extreme, as in the next case, it may be reflected in disease caused by the life pain behind the dependency. A patient of this kind is treated holistically in such a way that the physical pain is taken back to the dependency, which is taken back to the original pain of existence.

This history is also concerned with a major existential problem on how to relate to being the daughter of a prostitute. The person concerned was in the situation of being unwanted and sullied with shame from the start. How can you find self-esteem and worth against such a background? The patient had a hidden history, which was gradually confronted and put into place. At first it was highly embellished and idealized, then she realized the awful truth, and later an even deeper truth revealed itself, life's own truth, which resulted in resuming her course. The case illustrates how complex the situation often can be, since many things happen during life that it can be difficult to keep up with. The case history showed how disease is due to dependency, which in turn is due to an underlying existential emptiness. On the way through the course of the person's life, internal chaos is manifested as disease and external chaos as a miserable relationship, which ultimately in the case history was resolved in a necessary divorce.

> **Female, aged 38 years, where dependency is the actual problem underlying arthritis:** First conversation: The patient has a history of rheumatoid arthritis. She is on medication for this. She has three children, boys aged 8 and 12 years and a girl aged 6 years. Trained as auxiliary nurse, at present off work due to arthritis. She has previously had several operations on her knee for excrescences. Since the time she was very young, where she grew up with a hippie mother and does not know her father. Her mother was living in a lesbian relationship when she was small. She had to take responsibility for both her mother and her sister at an early age. She therefore had to be strong. "I'm disappointed," says the patient, and she looks very disappointed. She is disappointed with her husband, children, parents, family, friends, and everything. We work on this and talk about her use of power over those around her, who hurts her and others, and about her general malice towards herself and others. She finds it difficult to see this. EXERCISE for next time: Make a complete list of your power games. Another appointment in 2 weeks.
>
> Second conversation: Many thoughts about power games and about being domineering. Her father ended up in institutional care and her best friend fell into alcohol abuse and she is also carrying her husband. She is a mother to them all, but is tired of fulfilling this role, and everyone around her is dropping like rotting tree trunks. On the couch I (SV) work on her stomach, in which "she is not present" [I place one hand on the skin of her stomach and ask her to meet me there, while we talk together about topics relevant to the stomach]. She talks about her two childbirths, the first was terribly difficult, the second went fairly well. She finds it difficult to let go, to abandon herself, to be a woman. She has always been very masculine. It appears as though she allows herself to be a woman more on the couch. She is taking NSAID [drugs such as Ibuprofen] for her arthritis. She has started school. Next appointment in a week with the topic: letting go.
>
> Third conversation: "I am a woman," says the patient, and that does a lot for her, and she feels like kissing. She is living with a man she does not love, she is considering an affair, but I recommend that she should divorce straightaway so that the children come out of it unscathed. She is very anxious and sits clutching her stomach. She radiates "like a beautiful goddess" and talks about being sexually assaulted as a 6-year-old, how she was always afraid of men, and was very attractive as a teenager, before she became tough, masculine, and a driving force. We talk about her having to go back and feel the anxiety, but she is afraid of becoming fragile and being completely squashed, if that happens. Sometimes her legs turn to jelly. She will probably soon have to go for Rosen sessions[27]. Another appointment in 1 week. She must accept her anxiety, be fragile and emotional as a woman. She has the power in the relationship with her husband and therefore responsibility for it. She now has to live up to it. Her dependency on him must be broken and it is likely to be tough for her.

Fourth conversation: She has been through a lot since last session. Has suffered, been ill and vomiting. "It's dangerous to think about what I want from life," says the patient. She reveals her deep and shameful secret, which she has been battling with, alone for 7 years: that she is the result of rape. The story her mother has told her is as follows: Her mother was an escort and one day she had a very good-looking man as a client. It was in the sixties and at that time many girls earned money that way and they did not take it very seriously. The mother was very attracted to this man, who had apparently become hooked on her and eventually forced himself on her. She apparently gave in to him, let go, enjoyed it, and allowed herself to become pregnant; she did not have any regrets about it later, even though it did not fit in well with the social order. But the mother had lived in a lesbian relationship beforehand. The father left his visiting card and greatly regretted that he had not been able to control himself. On the couch, "I take her back to the time of conception", where we both look together at what actually happened. Very strong divine energy was present, from which sprang her purpose of life, she feels. She could not express this purpose in words today, but we will continue working on this. Another appointment in 1–2 weeks. Her arthritis is dormant. Today we worked on the content of the incident, which was extremely charged emotionally. It is not up to me to question the story; the patient's reality is what is concerned and that is what has to be handled if she is to be redeemed. The two main aspects focused on are the enormous sexual attraction that lie behind a case of rape – perhaps even mutual – and the subsequent moral condemnation. Gradually, as we work our way deeper into it, the polarization of the sexual pleasure and the shame is strengthened. In the patient's consciousness it emerges as an almost divine joining of the two sexual poles during the rape, which leads to her own creation and afterwards unbearable shame and guilt, which she shares with her mother. It is an extremely redeeming experience for the patient to have this dark side of her life confronted and integrated.

Fifth conversation: She phoned on Friday, having had too much to drink and said that she was deeply in love with me. Today we have talked about it and she is ashamed that she is unable to control herself. Much has opened up since the last session and she is full of feelings of all kinds. Her arthritis has disappeared completely, she has been able to do ballet on her toes again, which she had not been able to do for many years, and her pain has completely vanished. She herself thinks that her arthritis has been cured. We (SV and the patient) talk about how she fell in love, about it being "fine safe" to fall in love with a physician, who will not and cannot use it against her. She must accommodate her emotions for better or worse, until they calm down and fit into her new life. It is important not to shut herself off from her emotions again, because if she does, her arthritis will return. The emotions must be accommodated and developed, so that her emotionality and femininity are rehabilitated.

Sixth conversation: She has been to the rheumatology department and reports that the physician there told her that she had miraculously recovered – joints and cartilage have

> healed completely, no scarring can be seen on the X-ray and the radiologist has never seen that before, the patient reports. Her symptoms started when she had salmonella poisoning, was in such a poor state that she was unable to run, bicycle, and barely walk, after the physicians had saved her life. She felt terribly let down by her husband, who did not help her when it mattered. She now wants a divorce. Timeline therapy on this incident results in contact with a new incident, when the patient was 10 years old and bought trainers for her stepfather, who 2 days later falls off a ladder and only comes home after a year in intensive care, he died 6 years later from cancer. He was a good father of whom the patient was very fond. Scared of the ladder. Must never cry for her mother. Chain of anxiety? The patient promises to provide a copy of the hospital case record. EXERCISE 1: Write about the two incidents. EXERCISE 2: Tell your husband that you want a divorce, ask how long it will take him to find somewhere else to live, if appropriate find a place yourself. Suggestion for sharing daughter: every other weekend from Friday to Monday plus every Wednesday afternoon to Thursday afternoon plus first half of each holiday. If necessary, put this in writing for him.
>
> Seventh conversation: The patient says: “I am stupid, I am stupid, I am stupid,” and works on her duplicity, as she does not admit to her husband that she does not love him, that she does not like kissing him, that she does not like making love with him, that she does not fancy him, that she does not find him sexually attractive at all. There is no doubt about the need for them to separate, but it is very difficult for the patient to free herself of her dependency; she hides behind examinations, the children's needs, and so on. During the session, the patient became very disappointed and very angry with me and had an urge to strike me with flowers she had brought along to give me. We agreed, however, that she should come again. “You’re so false, the falseness runs down your teeth!” she says.
>
> Eighth conversation: She has lost her appetite and is not eating. On examination, she is very sore below the navel, “dependency relationship stomach”. She is about to separate, but her partner ignores her requests for him to move out, her 8-year-old son is in crisis. We talk about the patient having to review her superficial life and become genuine, deep, alive, and true. She is a facade person and it is very difficult for her to admit that she is in crisis; she is unable to accept the help that is offered. We talk about this. “I don’t know whether I would resist if someone held my head under water.” I suggest that we find a bucket at once and that makes her laugh. She is not considered to be suicidal.
>
> Ninth conversation: The patient is doing fine, she has found a new job, has developed really well, and feels sure that she will return to full health. /Finished/.

She was contacted by telephone 1 year later and she said: “I felt terribly cheated and helpless, which I think was the cause of the arthritis.” She said that the X-rays of her joints and lungs have again shown her to be completely healthy and the arthritic nodules have not returned. Her knee, which she was once told would never be repaired, now works perfectly. She says that she has changed many things in her life; she has divorced, she has changed her diet so that she eats less meat and animal fat, and has taken a part-time job 25 hours a week, which is what she is able to cope with now.

## DISCUSSION

The problem with dependency is that we are empty. Feeling this emptiness is almost unbearable. It is as though we would rather die than be empty. We have leaped out of ourselves and into the person we are dependent on. In psychodynamic terms, the dependency is a consequence of very severe neglect, which we repeat in relation to ourselves by completely omitting to feel what we need, where we are, and where we are to go. We are “in the other person” when it is a person we are dependent on, as in this case above, and barely sense ourselves or our own needs any longer. When we come “back home” to ourselves, we find a smoking ruin instead of our own good and orderly existence. There is a lot of clearing up to be done. The focus in the described case study was on the stomach and uterus (in the oriental tradition called the hara center), as it so often is with dependency.

The reward was reaped. The arthritis disappeared half way through the course of the treatment. This happened when she became willing to live with her emotional pains. The moment she did that, the problems moved out of her body, back into the emotions they come from, and the body was healed. She is still insecure and could still carry out maneuvers, where her defenses cleverly will take over. She can still get her arthritis back and she might perhaps simply find a new partner to be dependent on. She was over the worst after the last consultation and at the follow-up conversation 1 year afterwards, she was in good form with a new job and a good life, which appeared to be a stable improvement.

## CONCLUSION

The ways in which we escape hidden existential pains are multiple. The wide range of dependency states seems to be one of the most commonly used strategies. If the patient can be guided into the hidden existential pain to integrate it, in accordance with holistic process theory[9,10,11,12], dependency can be cured. The problem is that the patient must be highly motivated, sufficiently resourceful, and supported to want such a treatment, which is inherently painful. Often the family and surrounding world are suffering more than the dependent person himself, since the pattern of behavior the patient is dependent on makes him or her rather insensitive and unable to feel. If the patient is motivated, resourceful, and trusts in his physician, recovery from even a severe state of dependency is not out of reach, if the holistic medical tools are applied wisely. The patient must feel his old emotional pain, understand the source and inner logic of it, and finally “take to learning” and let go of negative attitudes and beliefs. In this way, fundamental existence can be healed and without the emotional suffering, the patient is no longer a slave of the dependency pattern, moving the attention away from the core of existence and life.

## ACKNOWLEDGMENTS

This study was supported by grants from IMK Almene Fond. The quality of life research was approved by the Copenhagen Scientific Ethical Committee, (KF)V.100.2123/91.

## BIOSKETCHES

**Søren Ventegodt, MD,** is the Director of the Quality of Life Research Center in Copenhagen, Denmark. He is also responsible for a Research Clinic for Holistic Medicine in Copenhagen and is a popular speaker throughout Scandinavia. He has published numerous scientific or popular articles and a number of books on holistic medicine, quality of life, and quality of working life. His most important scientific contributions are the comprehensive SEQOL questionnaire, the very short QoL5 questionnaire, the integrated QOL theory, the holistic process theory of healing, the life mission theory, and the Danish Quality of Life Research Survey, 1991–94 in cooperation with the University Hospital of Copenhagen and the late pediatric professor Bengt Zachau-Christiansen. E-mail: ventegodt@livskvalitet.org. Website: http://www.livskvalitet.org

**Mohammed Morad, MD**, is Specialist in Family Medicine, Lecturer in Family Medicine at the National Institute of Child Health and Human Development, Division of Community Health, Ben Gurion University of the Negev and the Medical Director of a large area clinic in the city of Beer-Sheva. He has publications on Bedouin health, health aspects, spiritual health, and aging in persons with intellectual disability, and is a presenter on topics such as health policy and services for the disadvantaged at national and international conferences. E-mail: morad62@barak-online.net

**Isack Kandel**, **MA, PhD**, is Senior Lecturer at the Faculty of Social Sciences, Department of Behavioral Sciences, the Academic College of Judea and Samaria, Ariel. During the period 1985–1993, he served as the Director of the Division for Mental Retardation, Ministry of Social Affairs, Jerusalem, Israel. E-mail: Kandeli@aquanet.co.il

**Joav Merrick, MD, DMSc,** is Professor of Child Health and Human Development affiliated with the Zusman Child Development Center, Division of Pediatrics and Community Health at the Ben Gurion University, Beer-Sheva, Israel; the Medical Director of the Division for Mental Retardation, Ministry of Social Affairs, Jerusalem; and the Founder and Director of the National Institute of Child Health and Human Development. He has numerous publications in the field of child and human development, rehabilitation, intellectual disability, disability, health, welfare, abuse, advocacy, quality of life, and prevention. Dr. Merrick received the Peter Sabroe Child Award for outstanding work on behalf of Danish Children in 1985 and the International LEGO-Prize (“The Children’s Nobel Prize”) for an extraordinary contribution towards improvement in child welfare and well being in 1987. E-mail: jmerrick@internet-zahav.net. Website: www.nichd-israel.com
